# The Curability of Insanity; as Illustrated by the Records of the Bloomingdale Asylum for the Insane

**Published:** 1848-02

**Authors:** 


*The Curability of Insanity; as Illustrated by the Records of the Bloomingdale Asylum for the Insane.* By Pliny Earle, M. D.

It appears that, from the time of the opening of the Asylum, June 16th, 1821, to December 31st, 1844, one thousand eight hundred and forty-one insane persons were received as patients.

The table subjoined exhibits the condition of those who had been discharged, and the numbers still remaining in the Asylum.

| | MALES. | FEMALES. | TOTAL. |
|---|---|---|---|
| Cured, | 408 | 264 | 672 |
| Much improved, | 58 | 46 | 104 |
| Improved, | 176 | 142 | 318 |
| Relieved, | 6 | 1 | 7 |
| Unimproved, | 2 | 1 | 3 |
| Discharged by request of friends, mostly unimproved, | 222 | 179 | 401 |
| Eloped, condition not stated, | 26 | 4 | 30 |
| Died, | 148 | 79 | 227 |
| Whole number discharged, | 1046 | 716 | 1762 |
| Remain, | 44 | 35 | 79 |
| Whole number admitted, | 1090 | 751 | 1841 |

One thousand seven hundred and sixty-two patients were discharged, of whom one thousand and forty six were men, and seven hundred and sixteen women. Of these, four hundred and eight men and two hundred and sixty-four women were cured, making a total of six hundred and seventy-two.

There were forty-two of the foregoing patients, twenty-three of whom were men and nineteen women, who after a short residence in the Institution, were discharged as follows:—

*Since reading the above case, an article by Dr. Blakeman, of New York, in the American Retrospect, has been pointed out to me, according to which he has employed this same treatment with success.

| | |
|---|---|
| Much improved, . . . . . . | 9 |
| Improved. . . . . . . . | 16 |
| Relieved, . . . . . . . | 1 |
| Discharged by request of friends. . . | 14 |
| Eloped. . . . . . . . . | 2 |

but after a brief absence, were re-admitted, and finally discharged cured. These cases should, most unquestionably, be added to the cures in the foregoing table.

It is now (August, 1847,) upwards of two years and a half since the close of the period embraced by these statistics. At that time, as will be perceived by the table, there remained in the Institution seventy-nine patients, who were here on their first admission. A large proportion of these were old, incurable cases, which had been in the Asylum for many years.

A sufficient time has now elapsed to test the curability of the few whose disease was of a more recent date. The subjoined list exhibits the present condition of the seventy-nine patients in question:—

| | MALES. | FEMALES. | TOTAL, |
|---|---|---|---|
| Discharged, cured, - - - | 4 | 6 | 10 |
| " much improved, - | 0 | 3 | 3 |
| " improved, - - | 8 | 3 | 11 |
| " unimproved, - - | 7 | 8 | 15 |
| Died, - - - - - - | 4 | 3 | 7 |
| Remaining, all incurable, - - | 21 | 12 | 33 |

The results in this table should also be added to the foregoing.

It is not unfrequently the case, that a patient who is progressing towards recovery is, through the anxiety of friends, prematurely removed, before a cure has been established. Eighteen cases of this kind, of which thirteen were discharged much improved, four improved, and one by the request of friends, the condition not stated, are known to have perfectly recovered soon after leaving. It is probable that there may have been more.

Our object being to ascertain, as nearly as possible, the curability of Insanity, it is very apparent that these cures, also, should be included in the original list. Indeed, it is but justice to the Institution that they should be included, inasmuch as, if they were cured by any system of treatment, it was that which was pursued here.

After these explanations, it appears that the cures were as follows:

| | MALES. | FEMALES. | TOTAL. |
|---|---|---|---|
| Discharged cured previously to December 31st, 1844, - - - | 408 | 264 | 672 |
| " cured subsequently, - - | 4 | 6 | 10 |
| " not cured on first admission, but cured on re-admission, - | 23 | 19 | 42 |
| Known to have recovered after discharge, - - - - - - | 6 | 12 | 18 |
| Aggregate, - | 441 | 301 | 742 |

As all the foregoiug cases which remain in the Institution are believed to be incurable, and as those who were discharged not cured have become widely scattered, and mostly lost sight of, it is probably that this table exhibits all, or very nearly all, who will ever be known to have been cured. This makes the per cent. of cures on all admissions 40.30. On the men it was 40.46, on the women 40.80.

Some authors believe that, of the insane, females are more curable than males. In these cases, however, as will be perceived, the comparative curability of the two sexes is somewhat in favor of the men.

It would hardly be justifiable to leave the subject of cures, without referring to a cause which has operated unfavorably upon the curability of the patients, taken as a whole, that have been received into this Institution. When the Asylum was first opened, fifty-two were transferred to it from the old Asylum. A great majority of these were chronic and incurable cases, which had been accumulating in that Asylum for many years. This fact will become evident if illustrated by the history of those cases, subsequent to their admission into this Institution, which is as follows:—

| | MALES. | FEMALES. | TOTAL. |
|---|---|---|---|
| The whole number was, - - - - | 32 | 20 | 52 |
| Discharged cured, - - - - - | 4 | 0 | 4 |
| " much improved, - - | 1 | 0 | 1 |
| " improved, - - - | 2 | 3 | 5 |
| " by request of frienes, mostly unimproved, - - - - - | 17 | 10 | 27 |
| Eloped, condition not stated, - - | 0 | 1 | 1 |
| Died, - - - - - - - | 2 | 4 | 5 |
| Remaining, Dec. 31st, 1844, - - | 6 | 3 | 9 |
| Total, - - - - - | 32 | 20 | 52 |

Thus it appears that only four of these cases were cured. This is equivalent to but 7.7 per cent.

The condition of the patients who were received from the Almshouse, in considerable numbers at a time, was very similar to those who were brought from the old Asylum. In the several transfers of patients which took place between the Almshouse and this Institution, several were brought here more than once. The whole number of first admissions was sixty-six, of whom twenty-nine were men and thirty seven women. Of these, only four men and twelve, a total of sixteen, were cured; and of all that were re-admitted, a recovery did not take place in a single case. Hence we have

| | MALES. | FEMALES. | TOTAL. |
|---|---|---|---|
| Admitted from old Asylum, - - | 32 | 20 | 52 |
| Admitted from Almshouse, - - | 29 | 37 | 66 |
| Total, - - | 51 | 57 | 118 |
| Of all these there were cured but - | 8 | 12 | 20 |

Now, substracting these cases from the whole number of admissions, and their cures from the whole number of cures,

| | | |
|---|---|---|
| We have the per cent. of cures in | men, - | 42.08 |
| Do. | women, - | 41.64 |
| Do. | both sexes, | 41.90 |

Physicians to Institutions for the Insane are frequently questioned in reference to the time necessary to effect a restoration in cases of insanity. The following table shows the term of residence in the Asylum of all the patients who were discharged cured:—

| | MALES. | FEMALES. | TOTAL. |
|---|---|---|---|
| Less than one month, - - | 4 | 31 | 76 |
| From one to two months, - - | 5 | 37 | 113 |
| From two to three months, - | 76 | 41 | 107 |
| From three to four months, - | 66 | 41 | 102 |
| From four to five months, - - | 61 | 25 | 54 |
| From five to six months, - - | 25 | 20 | 45 |
| From six to seven months, - | 29 | 17 | 46 |
| From seven to eight months, - | 10 | 12 | 22 |
| From eight to nine months, - | 8 | 6 | 14 |
| From nine to ten months, - - | 13 | 5 | 18 |
| From ten to eleven months, - | 5 | 7 | 12 |
| From eleven to twelve months, - | 11 | 4 | 15 |
| From one to two years, - - | 23 | 12 | 35 |
| Upwards of two years, - - | 7 | 6 | 13 |

The whole number who were in the Asylum less than three months each is two hundred and ninety-six. This is equivalent to forty-four in every hundred that were cured. The whole number from three to six months is two hundred and one, or thirty in every hundred. The whole number from six to twelve months is one hundred and twenty-seven, nearly nineteen in every hundred.

The whole number who were here upwards of one year each is forty-eight, or seven in every hundred.

The mean or average term of residence in the Asylum was, for the men, four months and twenty-seven days, and for the women five months and twenty-six days.

Setting aside the thirteen cases in which the persons were here more than two years each, the average time will be, for men four months and ten days, and for women, four months and twenty-five days.

The mean or average time of residence of both sexes inclusive is five months and eight days. Excluding the thirteen cases before mentioned, it is four months and sixteen days.

Many people who take their friends to an institution of this kind, appear to be impressed with the idea, that, if restoration be possible it can be effected in a few days, as if it were an ordinary fever. But insanity, particularly if reference be had to those cases alone which are sufficiently prolonged to induce their friends to remove them to an Asylum, is essentially a chronic disease, and even under the most skilful management, requires a considerable time for its removal, and the establishment of mental health. Were it possible always to induce the friends and guardians of patients to leave them at the Asylum a sufficient time, fully and satisfactorily to test their curability for the restorative means here employed, the recoveries would unquestionably be augmented, and that to no inconsiderable extent. At the Retreat, near York, England, where every patient is retained, if not cured, until all curative resources are exhausted, it is stated by the officers of that institution, that thirty-five per cent. of all the recoveries do not take place until the patients have been in the Asylum more than a year,—*New-York Annalist.*

